# Diffusion-weighted imaging and retinal oximetry as potential biomarkers of visual outcomes after optic neuritis

**DOI:** 10.1038/s41598-025-19331-w

**Published:** 2025-10-10

**Authors:** Pavel Hok, Jan Valošek, Tereza Králová, František Odstrčil, Martina Sapieta, Michal Král, Kruznev S. Nijhar, Anna Arkhipova, Monika Jasenská, Jan Mareš, Martin Šín

**Affiliations:** 1 Department of Neurology, Faculty of Medicine and Dentistry, Palacký University Olomouc, Olomouc, Czechia; 2 Department of Neurology, University Medicine Greifswald, Greifswald, Germany; 3 Functional Imaging Unit, Institute of Diagnostic Radiology and Neuroradiology, University Medicine Greifswald, Greifswald, Germany; 4 Department of Neurosurgery, Faculty of Medicine and Dentistry, Palacký University Olomouc, Olomouc, Czechia; 5 NeuroPoly Lab, Institute of Biomedical Engineering, Polytechnique Montreal, Montreal, QC Canada; 6 Department of Neurology, University Hospital Olomouc, Olomouc, Czechia; 7 Department of Radiology, University Hospital Olomouc, Olomouc, Czechia; 8 Department of Ophthalmology, University Hospital Olomouc, Olomouc, Czechia; 9 Department of Biomedical Engineering, University Hospital Olomouc, Olomouc, Czechia; 10 Department of Ophthalmology, Military University Hospital Prague, Prague, Czechia; 11 Present Address: Behavioural and Social Neuroscience, CEITEC – Central European Institute of Technology, Masaryk University, Kamenice 753/5, 62500 Brno, Czechia

**Keywords:** Optic neuritis, Multiple sclerosis, Biomarker, Diffusion weighted imaging, Retinal oximetry, Optical coherence tomography, Visual system, Multiple sclerosis

## Abstract

To elucidate the mechanisms influencing visual function recovery after optic neuritis (ON), this study employed a multicompartment diffusion weighted imaging (DWI) model to assess the role of optic radiation integrity and its relationship with retinal parameters, including automatic retinal oximetry and retinal nerve fiber layer (RNFL) thinning. Twenty-four patients with the first episode of acute unilateral ON were compared with 56 healthy volunteers with normal vision. Additionally, longitudinal analysis 3 and 6 months after ON was performed in 17 patients. Multivariate analysis of variance across baseline DWI metrics revealed a greater secondary partial volume fraction (f2) in patients. In the longitudinal analysis, a multivariate effect of time was observed only when adjusted for the affected side and time since onset; however, univariate post hoc tests were nonsignificant. An unadjusted model stratified according to clinical outcomes (best-corrected visual acuity [BCVA] and contrast sensitivity) indicated lower overall fractional anisotropy (FA) in patients with incomplete recovery. In the correlation analysis, baseline FA and oximetry (venous saturation and arteriovenous difference) predicted follow-up BCVA, whereas axial diffusivity predicted follow-up oximetry. In turn, baseline oximetry predicted follow-up RNFL thickness. In summary, DWI and retinal oximetry are both potential predictors of visual function outcomes after ON.

**Supplementary Information:**

The online version contains supplementary material available at 10.1038/s41598-025-19331-w.

## Introduction

Despite the general opinion that patients with optic neuritis (ON) typically achieve substantial recovery of visual function within several weeks, up to 40% of patients with ON diagnosed with multiple sclerosis (MS) exhibit abnormal best-corrected visual acuity (BCVA) or contrast sensitivity in the affected eye after 10 years^1^. Additionally, 60% of patients report persistent difficulties after ON, potentially impacting their quality of life^2^. Whereas standard treatment with intravenous methylprednisolone does not affect the long-term visual recovery^3^, early escalation therapy with plasma exchange seems to improve outcomes in patients with severe ON^4^. However, to identify patients who benefit from new treatment strategies, early biomarkers of functional recovery after ON are needed and are currently a matter of ongoing research^2^. Retinal imaging techniques such as optical coherence tomography (OCT) have been shown sensitive to retinal neuronal loss and to be predictive of resulting visual acuity^5^. Moreover, two OCT parameters, namely, the retinal nerve fiber layer (RNFL) and ganglion cell + inner plexiform layer (GCIPL), have been identified as reliable biomarkers of MS progression^6,7^. However, retinal thinning develops gradually after acute ON and its assessment may be affected by initial inflammatory swelling, therefore, more immediate disease biomarkers are required^8^.

Besides OCT, complementary information on underlying processes can be obtained using automatic retinal oximetry, a non-invasive retinal imaging technique to assess retinal oxygen metabolism with good reproducibility^9^. It has demonstrated increased retinal venular oxygen saturation (VS) and lower arteriovenous difference (AVD) in patients with MS with ON history^10^ but not in those without ON^11^, putatively reflecting reduced oxygen uptake associated with retinal atrophy^10^. In the acute stage of ON, oximetry has further revealed increased arteriolar oxygen saturation (AS), which preceded the decrease in retinal oxygen consumption 6 months after ON associated with the RNFL thinning^12,13^. Retinal oximetry has thus potential to reflect both the acute local metabolic effects of inflammation and subsequent neurodegeneration^12,13^.

New potential biomarkers should also reflect retrochiasmal structural abnormalities, as optic nerve inflammation cannot fully account for the decline in visual function in patients with MS^14^. Therefore, combination of retinal assessments with additional magnetic resonance imaging (MRI)-based diagnostic tools, such as brain diffusion weighted imaging (DWI) with diffusion tensor imaging (DTI)^15^ might provide further insight into the interaction between retinal damage and white matter integrity of the optic pathway. In MS, fractional anisotropy (FA) of the optic radiation (OR) has been shown to correlate with the RNFL, low contrast visual acuity^16^ or visual evoked potential (VEP) latency^17^. More recently, axial diffusivity (AD) of the OR within 1 month after onset of ON was shown to be predictive of the RNFL and VEP after 12 months^18^. However, parameters obtained from more complex diffusion models^19,20^ were shown to be more sensitive to underlying pathological processes even at disease onset^21^ and better characterize clinical outcomes in MS^22^. Similarly, primary and secondary partial volume fractions (f1 and f2) from the ball-and-stick multicompartment model^23^, which represent FA-like measures estimated for non-colinear axonal populations, have recently been shown to be more sensitive to tissue damage than conventional DTI parameters at the spinal level^24,25^. It is yet unclear whether these additional DWI parameters are more sensitive to axonal damage in ON and whether they are associated with visual function outcomes. Furthermore, the associations between DWI and retinal oximetry parameters have not yet been evaluated.

Hence, we aimed to investigate the relationship between visual functional impairment and the integrity of the OR, as well as the interaction between the integrity of the OR and the processes occurring in the retina at different time-scales (early metabolic abnormalities in oximetry or late cell loss in OCT) in a single longitudinal study.

Specifically, we hypothesized that (1) DWI parameters (FA, mean diffusivity [MD], axial diffusivity [AD], radial diffusivity [RD], f1, f2) of the OR on each side would differ between patients with ON and healthy controls (HCs) at baseline (M0). (2) DWI parameters of the OR on each side change across follow-up (M0, month 3 [M3], and month 6 [M6]) in (a) all patients and especially in those (b) with incomplete recovery (BCVA at M6 < 20/20 or contrast sensitivity using Pelli‒Robson score^26^ at M6 < 1.65 log units of the affected eye [AE]). (3) DWI parameters correlate with (a) BCVA, (b) Pelli‒Robson score, (c) RNFL, and (d) retinal oximetry parameters (AS; VS, and AVD) of the AE at M0, M3, and M6. (4) DWI parameters at M0 predict (a) BCVA, (b) Pelli‒Robson, (c) RNFL, (d) AS, VS, and AVD of the AE at M6 after ON.

The following auxiliary hypotheses were tested in patients with ON: (5a) RNFL, (5b) AS, VS, and AVD of the AE correlate with BCVA and Pelli‒Robson AE at each time point; (6a) RNFL, (6b) AS, VS, and AVD of the AE at M0 predict ipsilateral BCVA and Pelli‒Robson score at M6; (7) AS, VS, and AVD of the AE predict RNFL at M6; and (8) DWI parameters correlate with lesion load at each time point.

## Methods

### Participants

Consecutive patients with a first episode of ON were recruited between 2019 and 2022 in a neurological department at a tertiary-level hospital for this longitudinal study. The inclusion criteria for patients were as follows: age 18–65 years, history of acute unilateral ON that occurred during the previous 8 weeks with no systemic steroid treatment prior to current admission, fulfilling the criteria for clinically isolated syndrome (CIS) or relapse-remitting MS as evaluated by a treating neurologist on the basis of the 2017 revised McDonald criteria^27^. Furthermore, two HCs were recruited per patient. The inclusion criteria for HCs were as follows: no history of any neurological or ophthalmologic conditions and no signs of motor disability upon enrollment and sex and age matched to patients. The exclusion criteria for both patients and HCs were a history of diabetes; other ocular diseases, including glaucoma, cataracts, and age-related macular degeneration; refractive errors greater than or equal to ± 6 dioptres; and a history of vitrectomy. An additional exclusion criterion from the analysis for patients was recurrent ON. Recruitment was carried out as a part of a multimodal longitudinal imaging study, and no dedicated sample size estimation was performed a priori for the hypotheses evaluated here.

The study was carried out in accordance with the World Medical Association Declaration of Helsinki. Written informed consent was obtained from all participants prior to their inclusion in the study, and the study was approved by and conducted in compliance with the Ethics Committee of the University Hospital and the Faculty of Medicine and Dentistry, Palacký University in Olomouc, approval number NV19-06-00216.

### Assessment schedule

At the first visit (M0), each participant underwent standard ophthalmologic evaluation to rule out conditions other than ON, an MRI session, automatic retinal oximetry, and OCT. Patients were scheduled for follow-up visits 3 (M3) and 6 months (M6) after the first visit, and all assessments from the M0 visit were repeated. To avoid performance bias, all patients additionally underwent standard clinical neurological assessments and received standard treatment according to national guidelines and at the discretion of the treating neurologist, including disease-modifying therapy in the indicated cases. All assessments as well as definitions of variables of interest and potential confounders are provided in Supplementary Table 1.

### Clinical assessments

All participants were screened for a history of any neurological or ophthalmologic condition. Handedness was initially assessed in all participants using the Edinburgh Handedness Inventory^28^. Ocular dominance was established at each visit via a hole-in-card procedure. The BCVA was measured using an Early Treatment Diabetic Retinopathy Study (ETDRS) visual acuity chart according to the standard protocol^29^. Contrast sensitivity was assessed using the Pelli‒Robson test^26^. A BCVA of < 20/20 and/or a contrast sensitivity score of < 1.65 log units at M6 were considered abnormal outcomes. Patients with MS underwent standard neurological examination, providing disability assessment using the Expanded Disability Status Scale (EDSS)^30^.

### Optical coherence tomography (OCT)

The RNFL measurement was performed using high resolution spectral domain OCT (SD-OCT) combining OCT technology with a confocal scanning laser ophthalmoscope (Spectralis, software version 4.0.3.0; Heidelberg Engineering, Heidelberg, Germany) as previously described^13^. All the RNFL scans were acquired after pupil dilation, allowing a detailed differentiation of the retinal layers. Each eye was scanned repeatedly within one session until at least 3 high-quality RNFL scans were obtained for further analysis.

### Automatic retinal oximetry

Retinal oximetry was carried out using an Oxymap oximeter (Oxymap ehf., Reykjavik, Iceland). A standardized acquisition technique was employed, with the full description provided elsewhere^13,31^. Image analyses were performed according to a standardized protocol (Oxymap protocol for acquisition and analysis of Oxymap T1 oximetry images, version November 21, 2013; Oxymap ehf.) using the software Oxymap Analyzer (version 2.5.2, Oxymap ehf., Reykjavik, Iceland). The arteriovenous difference (AVD) was calculated as the difference between the retinal arteriolar oxygen saturation (AS) and the retinal venular oxygen saturation (VS)^32^.

### MRI data acquisition

Imaging data were acquired using a 1.5 T (Siemens Aera, Erlangen, Germany) scanner with a 20-channel head/neck coil. The subject’s head was immobilized with cushions to assure comfort and minimize head motion. The imaging protocol included a high-resolution three-dimensional isotropic T_1_-weighted scan using magnetization-prepared rapid acquisition with gradient echo (MPRAGE) sequence with repetition time (TR) = 2460 ms, echo time (TE) = 3.65 ms, inversion time (TI) = 1240 ms, flip angle (FA) = 8°, 224 sagittal slices with a 0.5-mm gap, field of view (FOV) = 256 × 256 mm with 1 mm isotropic resolution, parallel acquisition techniques (PAT) factor = 2, fat suppression enabled; an in-plane fluid-attenuated inversion recovery (FLAIR) image with TR = 5000 ms, TE = 335 ms, TI = 1800 ms, FA = 120°, 176 sagittal slices, in-plane FOV = 224 × 256 mm, resolution 1 mm isotropic, PAT factor = 2; and a single-shot spin‒echo echo-planar imaging (EPI) diffusion-weighted sequence (TR = 6600 ms per slice, TE = 92 ms, FA = 90°, b = 1000 s/mm^2^, bandwidth 888 Hz/Px, 64 directions covering the full sphere, 5 reference B_0_ images, FOV = 260 × 260 mm, 40 axial slices, voxel size 2 mm isotropic, and no gap between slices, PAT factor = 2, partial Fourier = 6/8, anterior–posterior phase encoding) covering the occipital and temporal lobes. A set of 6 reference B_0_ images with inverted phase encoding (i.e., posterior-anterior phase encoding) was acquired for off-line correction of B_0_ off-resonance geometrical distortions.

### MRI data analysis

DWI data were processed using FMRIB Software Library (FSL) version 6.0.5^33^ to correct for motion, susceptibility distortions and eddy current distortions^34,35^. The conventional DTI model^15^ and multicompartment ball-and-stick model accounting for crossing fibers^23^ were subsequently estimated to extract microstructural parameters (FA, MD, AD, and RD for the DTI model and f1 and f2 for the ball-and-stick model).

A two-step registration of a skull-stripped diffusion-weighted image to a standard-space MNI152 structural image^36^ via the individual structural image (MPRAGE) was carried out, providing a nonlinear warping field between the diffusion space and MNI152 standard space. Next, the XTRACT tool^37–39^ was used to perform probabilistic tractography of the left and right ORs (independent of the affected side) via standardized masks warped into diffusion space, hence, the tractography was performed in native space. Masks for tractography consisted of a seed mask placed in the lateral geniculate nucleus and a target mask in a coronal plane through the anterior part of the calcarine fissure. The exclusion mask consisted of an axial block of the brainstem, a coronal plane anterior to the seed, and a coronal block directly posterior to the seed^39^. Microstructural parameters were extracted for each participant and each tract after a 5% threshold using the xtract_stats tool, effectively extracting parameters from the OR core^39^.

### Lesion load assessment

The individual T_2_-weighted lesion load was calculated using the Lesion Segmentation Tool version 3.0.0 (LST, www.statistical-modelling.de/lst.html) with a lesion prediction algorithm^40^. The resulting lesion maps were binarized at 0.5 threshold. For each participant, the volume fraction of the OR on each side affected by T_2_-weighted lesions was evaluated.

### Statistical analysis

First, normality was assessed for continuous variables using the Shapiro–Wilk test. Next, potential differences in demographic (age, sex, handedness, ocular dominance), clinical (BCVA, contrast sensitivity, RNFL, retinal AS, VS, and AVD), and auxiliary imaging variables (lesion load) were assessed: comparison patients vs. HCs and/or comparisons M0 vs. M3, M0 vs. M6, and M3 vs. M6 where applicable. Dichotomous variables were analyzed using Fisher’s exact test for 2 × 2 contingency tables (Pearson chi-square in the case of non-dichotomous nominal variables). The remaining non-normally distributed variables were compared between groups using the Mann–Whitney U test, whereas within-group comparisons were carried out using the Wilcoxon signed rank test.

Hypotheses 1 and 2 were tested using doubly multivariate analysis of variance (multivariate ANOVA, or MANOVA) with DWI parameters (FA, MD, AD, RD, f1, f2) extracted from the left and from the right ORs as dependent variables. In each model, the hemisphere (representing the left or the right OR independent of the affected side) was included as a within-subject factor. After confirming that the dependent variables did not deviate significantly from the normal distribution, a bivariate Pearson correlation for each pair of variables was calculated to assess potential multicollinearity (|*r*|> 0.9). This was the case for correlations between FA and f1, RD and f1, and partly between FA and RD. For the sake of comparability with previous studies reporting FA, the two remaining offending variables (f1 and RD) were discarded from the main analyses. However, to evaluate how the results were affected by this arbitrary choice, we conducted a sensitivity analysis, in which the MANOVA was additionally repeated after excluding FA and RD while including f1.

For Hypothesis 1, patient and HC data at M0 were compared with group as a between-subject factor, with no demographic confounders assumed. For Hypothesis 2a, patient data for M0, M3, and M6 were compared with time as a within-subject factor, again assuming no confounder. For Hypothesis 2b, abnormal outcome at M6 (BCVA < 20/20 and/or Pelli‒Robson score < 1.65 log of the AE) was added as a between-subject factor. Details on MANOVA procedure and sensitivity analyses with models adjusted for demographic and clinical confounding factors are provided in Supplementary Methods.

For the remaining hypotheses, bivariate correlations were assessed within the patient group using the two-tailed Spearman rank correlation coefficient. First, the relationships between the DWI parameters (FA, MD, AD, RD, f1, f2) and ophthalmological assessments (BCVA, Pelli‒Robson score, RNFL, AS, VS, AVD) were evaluated at the corresponding time points (Hypothesis 3). Next, the correlations between the baseline DWI parameters (M0) and the follow-up ophthalmology assessments (M6) were calculated (Hypothesis 4). Correlations for the main hypotheses were considered significant at *p* < 0.05 with additional Bonferroni‒Holm correction for the number of independent variables. For auxiliary Hypotheses 5–7, see description in Supplementary Methods.

All analyses were performed in SPSS Statistics 30 (IBM, Armonk, NY, USA, https://www.ibm.com/analytics/spss-statistics-software) with an alpha significance level of *p* < 0.05. Missing data were excluded pairwise.

### Additional post hoc analyses

Owing to the low variability of BCVA and contrast sensitivity score at M6, an additional receiver operating characteristic (ROC) analysis was carried out for the prediction of aggregate abnormal outcomes at M6 (BCVA < 20/20 and/or Pelli‒Robson score < 1.65 log of the AE) with the DWI parameters (FA) and ophthalmological assessments (VS) significantly associated with any of the outcome measures.^41^ Uncorrected asymptotic significance at *p* < 0.05 was considered significant for the null hypothesis area under curve (AUC) = 0.5, with the paired-sample AUC difference considered significant at *p* < 0.05 under the null hypothesis AUD difference = 0.

## Results

### Study sample

In total, 33 consecutive patients with ON and 68 HCs were enrolled. Subsequently, 12 HCs and 8 patients were excluded due to missing or corrupt data (for more details on exclusion, dropout and number of patients and HCs entering each analysis, see Supplementary Fig. S1). All 25 included patients were classified as CIS at the time of M0. The demographic parameters and group comparisons of the included subjects are provided in Table 1. Overall, there were no significant differences between the patient and HC groups. Individual clinical characteristics of included patients are provided in Table 2.

**Table 1:** Demographic characteristics and group comparisons.

	Patients (*n* = 25)	HC (matched) (*n* = 25)	*p*	HC (*n* = 56)	*p*
Age [years]*	32.8 ± 14.3	30.3 ± 9.6	0.691^†^	29.2 ± 5.8	0.481^†^
Sex (female/male)	20/5	20/5	1.000^‡^	37/19	0.293^‡^
Ocular dominance (L/R/missing)	8/17/0	8/17/0	1.000^§^	13/40/3	0.536^§^
Handedness LI*	0.9 ± 0.3	0.8 ± 0.3	0.889^†^	0.8 ± 0.3	0.765^†^
Affected side (L/R)	6/19	N/A	N/A	N/A	N/A

*HC* healthy controls, *L* left, *LI* (Edinburgh Handedness Inventory) laterality index, *N/A* not applicable, *R* right.

*Median ± interquartile range; ^†^Mann‒Whitney U test; ^‡^Fisher’s exact test; ^§^Pearson Chi-Square (exact).

**Table 2:** Clinical patient characteristics.

*n*	Age range	Sex	Affected eye	Time onset-MRI M0 [d]	Time corticoids-MRI M0 [d]*	BCVA (decimal)			Pelli‒Robson [log units]			Signs of ION	Relapses	DMT	Dropout
						M0	M3	M6	M0	M3	M6		*n*	M6	
1	21–25	f	R	7	1	1.00	1.00	N/A	N/A	N/A	N/A	no	0	None	WD M6 – pregnancy
2	26–30	f	R	28	− 1	0.80	0.80	1.00	N/A	N/A	N/A	no	0	None	
3	≤ 20	f	L	4	0	0.16	N/A	1.00	N/A	N/A	N/A	no	0	None	
4	21–25	f	R	7	− 1	0.03	0.80	0.63	N/A	N/A	1.65	yes	1	None	
5	36–40	f	R	54	− 1	0.32	0.40	0.25	N/A	N/A	N/A	no	0	None	
6	31–35	m	L	15	− 1	0.40	N/A	1.00	N/A	N/A	1.95	no	1	None	ON relapse
7	21–25	f	R	4	− 2	0.63	1.00	N/A	N/A	1.80	N/A	no	0	IFN-β	WD M6 – pregnancy
8	21–25	f	L	6	0	1.00	1.00	1.00	N/A	N/A	1.95	no	0	IFN-β	
9	41–45	f	L	13	7	0.20	0.50	0.50	N/A	1.35	1.35	yes	1	N/A	ON relapse
10	36–40	f	L	32	1	0.63	0.00	0.00	1.20	0.30	0.00	yes	1	None	ON relapse
11	41–45	f	L	9	0	0.70	N/A	N/A	0.60	N/A	N/A	yes	N/A	None	WD M3 – no reason
12	36–40	f	R	21	5	1.00	1.00	1.00	1.65	1.80	1.95	no	0	TFL	
13	21–25	f	R	7	2	0.80	0.80	1.00	1.50	1.35	1.35	no	0	IFN-β	
14	31–35	m	L	13	3	0.80	1.00	1.00	1.50	1.80	1.80	no	0	IFN-β	
15	31–35	m	L	13	1	1.00	1.00	1.00	1.95	1.95	1.95	yes	0	TFL	
16	≤ 20	f	L	11	0	0.03	0.80	1.00	0.60	1.95	1.95	yes	0	IFN-β	
17	26–30	f	L	11	7	1.00	1.00	1.00	1.95	1.95	1.95	yes	0	TFL	
18	26–30	f	L	22	− 3	1.00	1.00	1.00	1.50	1.95	1.95	no	0	None	
19	36–40	f	L	7	4	0.12	1.00	1.00	0.60	1.60	1.65	no	0	TFL	Missing MRI at M6
20	≤ 20	m	L	10	3	0.32	1.00	0.80	0.75	1.65	1.95	no	0	OFA	
21	46–50	f	L	7	3	0.05	1.00	1.00	0.00	1.80	1.95	no	0	IFN-β	Missing MRI at M0
22	31–35	m	L	2	0	0.80	1.00	1.00	1.05	1.95	1.95	yes	0	IFN-β	
23	26–30	f	L	11	2	0.63	1.00	1.00	1.35	1.80	1.95	no	2	OFA	
24	41–45	f	R	54	17	1.00	1.00	1.00	1.65	1.65	1.65	no	0	OFA	
25	36–40	f	R	11	1	0.03	0.05	0.05	0.15	0.30	0.45	yes	0	TFL	

*DMT* disease modifying therapy, *f* female, *IFN-β* interferon β1a, *ION* intraocular neuritis, *L* left, *m* male, *M0* month 0 (baseline), *M3* month 3, *M6* month 6, *N/A* not applicable, *OFA* ofatumumab, *ON* optic neuritis, *R* right, *rON* recurrent optic neuritis, *RRMS* relapsing–remitting multiple sclerosis, *SD* standard deviation, *TFL* teriflunomide, *yrs* years, *WD* withdrawn.

*Positive values indicate corticoid treatment initiated before magnetic resonance imaging.

### Clinical assessment

Both BCVA and contrast sensitivity in the AE were significantly lower in patients at baseline (median decimal BCVA = 0.67; median Pelli‒Robson score = 1.28 log units) than in HCs with normal vision (Table 3). While contrast sensitivity significantly improved at each time point (median Pelli‒Robson score was 1.80 and 1.95 log units for M3 and M6, respectively), a significant improvement in BCVA was observed only between M0 and M3 (median BCVA was 1.00 for both M3 and M6), as shown in Table 3 for statistical comparisons. In total, 5 patients (26.3% of the 19 patients included in the follow-up analysis) still presented with abnormal vision of the AE at M6, i.e., BCVA < 20/20 or Pelli‒Robson score < 1.65 log.

**Table 3:** Visual function and retinal imaging, group comparisons.

		Patients—affected eye					HC—random eye			*p* v. PwON^†^
		Median	IQR	*n*	*p* v. M3* (*n*)	*p* v. M6* (*n*)	Median	IQR	*n*	
Decimal BCVA	M0^‡^	0.63	0.82	25	**0.003** (20)	**0.002** (19)	1.00	0.00	56	** < 0.001**
	M3^§^	1.00	0.20	20		0.518 (18)				** < 0.001**
	M6^§^	1.00	0.00	19						**0.001**
Pelli‒Robson score	M0^‡^	1.28	1.01	16	**0.006** (14)	**0.004** (14)	1.95	0.00	39	** < 0.001**
	M3^§^	1.80	0.30	15		**0.024** (14)				** < 0.001**
	M6^§^	1.95	0.30	16						0.092
RNFL [μm]	M0^‡^	102	23	25	**0.002** (20)	**0.003** (19)	N/A	N/A	0	N/A
	M3^§^	96.5	24	20		**0.047** (18)				N/A
	M6^§^	87.0	23	19						N/A
Retinal AS [%]	M0^‡^	98	4	23	0.931 (17)	0.391 (18)	97	3	56	0.125
	M3^§^	98	3	19		0.806 (17)				0.309
	M6^§^	98	7	19						0.063
Retinal VS [%]	M0^‡^	66	8	23	0.434 (17)	0.571 (18)	65	7	56	0.378
	M3^§^	65	9	19		0.962 (17)				0.898
	M6^§^	67	10	19						0.893
Retinal AVD [%]	M0^‡^	31	10	23	0.619 (17)	0.631 (18)	32	6	56	0.725
	M3^§^	32	8	19		0.669 (17)				0.669
	M6^§^	33	8	19						0.709

*Wilcoxon Signed Rank Test; ^†^Mann‒Whitney U test; ^‡^all included subjects; ^§^PwON included in follow-up analyses (M3 sample, *n* = 21).

*AS* arteriolar (oxygen) saturation, *AVD* arterio-venous difference, *BCVA* Best-Corrected Visual Acuity, *IQR* interquartile range, *L* left, *M0* month 0, *M3* month 3, *M6* month 6, *N/A* not applicable, *PwON* patients with optic neuritis, *R* right, *RNFL* retinal nerve fiber layer, *VS* venular (oxygen) saturation.

We observed a significant gradual decline in median RNFL thickness in the AE, decreasing from 101.5 μm at M0 to 87.0 μm at M6 (Table 3). In contrast, we found no difference in retinal oxygen saturation levels between the patients and HCs (AE vs. random eye) at baseline and observed no change in the AE in patients during follow-up (Table 3).

### Lesion load assessment

The median ± interquartile range (IQR) of the T2-weighted lesion load for all included patients at baseline (n = 25) was 175 ± 632 mm^3^; for patients included in the follow-up analysis, it was 192 ± 760 mm^3^ at M0, 156 ± 471 mm^3^ at M3, and 248 ± 645 mm^3^ at M6. No paired-sample differences in lesion load between sessions were detected (uncorrected *p*_*M0–M3*_ = 0.167, *p*_*M0–M6*_ = 0.278, *p*_*M3–M6*_ = 0.868, Wilcoxon signed rank test). The individual lesion volume fraction in the OR ranged from 0 to 4.0% (median = IQR = 0% for both the left and right ORs at each time point); hence, complete fiber tracts were assessed in the statistical analysis.

### Differences in DWI at baseline

**Fig. 1:**
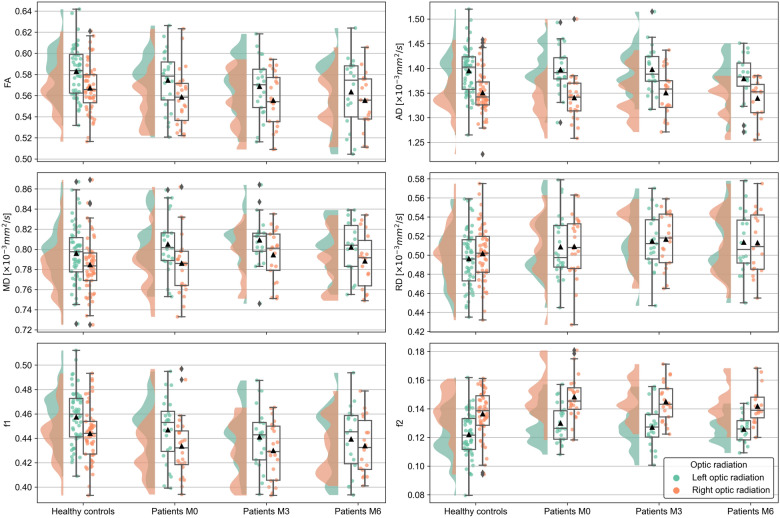
Diffusion-weighted imaging (DWI) parameters by group and visit. The raincloud plots in all panels show distributions as well as individual values for fractional anisotropy (FA), axial, mean and radial diffusivity (AD, MD, RD, respectively), and primary and secondary partial volume fractions (f1 and f2, respectively) in healthy controls and patients with optic neuritis at months 0, 3, and 6 (M0, M3, and M6, respectively). Data from the left (green) and right (red) optic radiations are displayed separately. The embedded box plots additionally provide medians, quartiles and outliers, whereas the mean is indicated by a triangle.

In total, 24 patients and 56 HCs were eligible for analysis of group differences in DWI parameters at baseline (Hypothesis 1). Summary statistics are provided in Supplementary Table 2. In the unadjusted model, MANOVA yielded significant multivariate effects of hemisphere and group (i.e., difference between patients with ON and HCs). For the factor hemisphere, post hoc ANOVA confirmed that higher FA, MD and AD values and lower f2 values were detected in the left OR than in the right OR independent of group membership. In contrast, group differences were only detected for f2, indicating higher f2 in patients than in HCs (see Table 4 and Fig. 1).

**Table 4:** MANOVA and post hoc ANOVA for group comparison at baseline.

MANOVA							ANOVA				
Model	Factor	Wilk’s λ	df*	*F*	*p*	Partial *η*^*2*^	Variable	df*	*F*	*p*^†^	Partial *η*^*2*^
Unadjusted 24 patients vs. 56 HCs	Group	0.842	4, 75	3.526	0.011	0.158	FA	1, 78	2.503	0.118	0.031
							MD	1, 78	0.639	0.427	0.008
							AD	1, 78	0.148	0.702	0.002
							f2	1, 78	8.799	**0.004**	0.101
	Hemisphere	0.310	4, 75	41.729	** < 0.001**	0.690	FA	1, 78	34.210	** < 0.001**	0.305
							MD	1, 78	49.453	** < 0.001**	0.388
							AD	1, 78	138.484	** < 0.001**	0.640
							f2	1, 78	81.230	** < 0.001**	0.510
	Group × hemisphere	0.930	4, 75	1.415	0.237	0.070					
Adjusted 24 patients vs. 56 HCs	Group	0.905	4, 72	1.894	0.121	0.095					
	Hemisphere	0.937	4, 72	1.220	0.310	0.063					
	Age	0.935	4, 72	1.260	0.294	0.065					
	Sex	4.798	4, 72	0.790	**0.002**	0.210	FA	1, 75	2.752	0.101	0.035
							MD	1, 75	0.415	0.521	0.006
							AD	1, 75	4.185	0.044	0.053
							f2	1, 75	16.643	** < 0.001**	0.182
	Group × sex	0.968	4, 72	0.599	0.665	0.032					
	Group × hemisphere	0.951	4, 72	0.921	0.457	0.049					
	Hemisphere × age	0.991	4, 72	0.163	0.956	0.009					
	Hemisphere × sex	0.994	4, 72	0.117	0.976	0.006					
	Group × hemisphere × sex	0.991	4, 72	0.169	0.953	0.009					
Unadjusted 24 patients vs. 24 HCs	Group	0.771	4, 43	3.199	**0.022**	0.229	FA	1, 46	4.780	0.034	0.094
							MD	1, 46	0.533	0.469	0.011
							AD	1, 46	1.266	0.266	0.027
							f2	1, 46	7.795	**0.008**	0.145
	Hemisphere	0.303	4, 43	24.704	** < 0.001**	0.697	FA	1, 46	28.181	** < 0.001**	0.380
							MD	1, 46	41.767	** < 0.001**	0.476
							AD	1, 46	93.242	** < 0.001**	0.670
							f2	1, 46	48.213	** < 0.001**	0.512
	Group × hemisphere	0.872	4, 43	1.572	0.199	0.128					

*AD* axial diffusivity, *ANOVA* analysis of variance, *df* degrees of freedom, *f2* secondary partial volume fraction, *FA* fractional anisotropy, *HCs* healthy controls, *MANOVA* multivariate ANOVA, *MD* mean diffusivity.

*Order: hypothesis df, error df; ^†^Bonferroni-corrected significance level *p* < 0.0125 for *n* = 4 marked in bold.

**Fig. 2:**
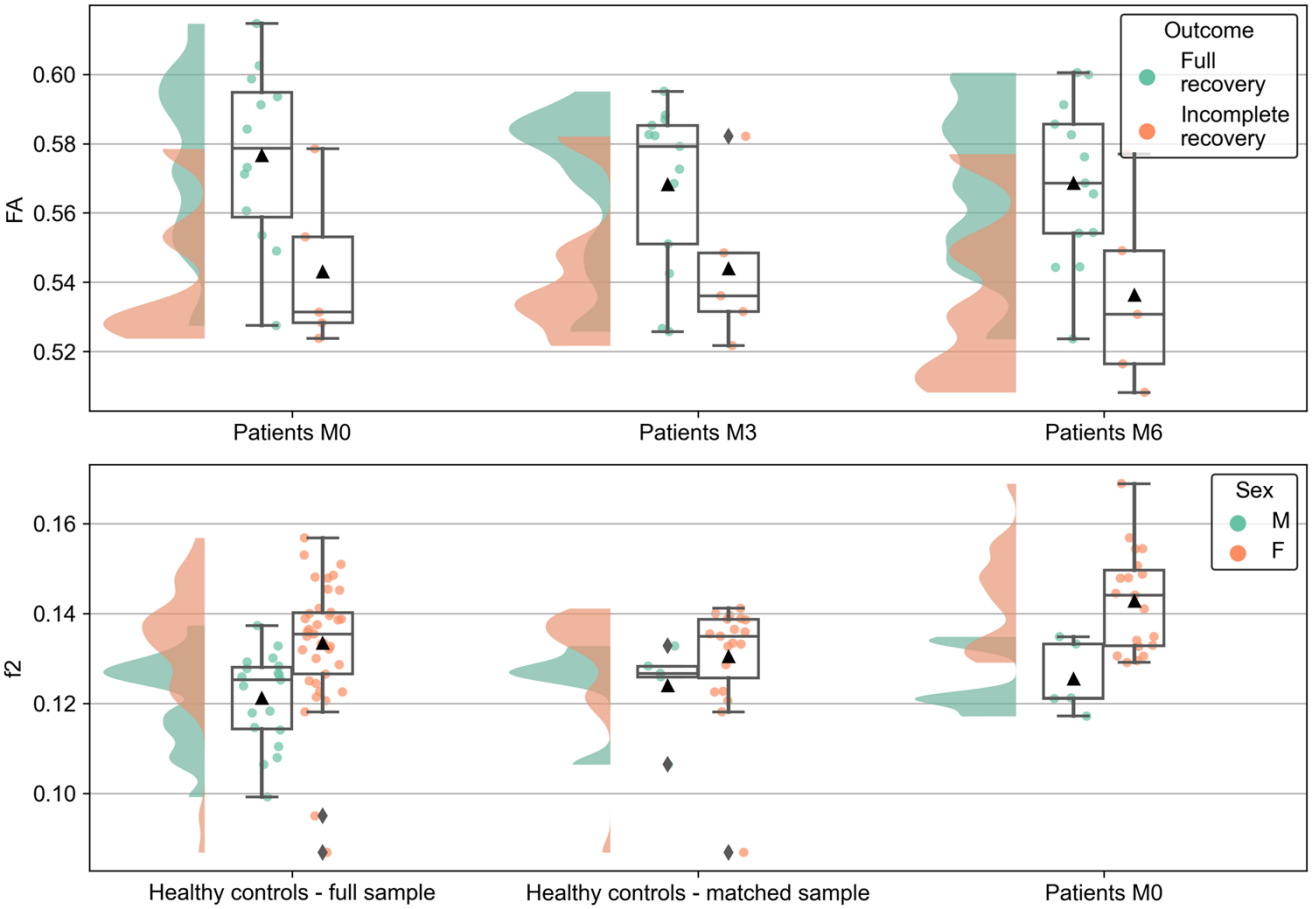
Diffusion-weighted imaging (DWI) parameters grouped by outcomes and sex. The raincloud plots show distributions as well as individual values for fractional anisotropy (FA, top panel) and for the secondary partial volume fraction (f2, bottom panel). In both panels, values for the right and left hemisphere were averaged. In the top panel, patient data for months 0, 3, and 6 (M0, M3, and M6, respectively) are sorted according to complete (green) or incomplete (red) visual function recovery, defined as best-corrected visual acuity (BCVA) < 20/20 and/or Pelli‒Robson score < 1.65 log of the affected eye at M6. In the bottom panel, data from healthy controls (both the full sample with n = 56 and the matched sample with n = 24) and patients at M0 were grouped by sex (green—left for males [M] and red—right for females [F]). As in Fig. 1, the embedded box plots show the medians, quartiles and outliers, whereas the mean is indicated by a triangle.

The sensitivity analyses for the group comparison provided mixed results. An unadjusted model with 24 matched HCs confirmed significant multivariate effects of group and hemisphere (Table 4). However, in a model with all 56 HCs adjusted for sex and age, neither factor group nor hemisphere remained significant at the multivariate level. Instead, a significant multivariate effect of sex was observed, reflecting a lower f2 in male than in female participants (see Table 4 and Fig. 2). Finally, the corresponding analyses with f1 (instead of FA) yielded results virtually identical to the main results with FA (see Supplementary Table 3).

### Longitudinal changes in DWI

In total, 17 patients were eligible for the longitudinal analysis (Hypothesis 2a). In the unadjusted model, MANOVA yielded significant multivariate effects for the hemisphere (i.e., difference between the left and right ORs) and interaction time × hemisphere. The post hoc tests for time × hemisphere were non-significant (Table 5); hence, the main effect of hemisphere was examined, yielding significant post hoc *F* tests for MD, AD, and f2 but not for FA (Table 5 and Fig. 1).

**Table 5:** MANOVA and post hoc ANOVA for longitudinal assessment in patients, irrespective of outcome.

MANOVA							ANOVA				
Model	factor	Wilk’s λ	df*	*F*	*p*	Partial *η*^*2*^	Variable	df*	*F*	*p*^†^	Partial *η*^*2*^
Unadjusted	Time	0.580	8, 9	0.815	0.608	0.420					
	Hemisphere	0.219	4, 13	11.599	** < 0.001**	0.781	FA	1, 16	5.151	0.037	0.244
							MD	1, 16	15.308	**0.001**	0.489
							AD	1, 16	38.641	** < 0.001**	0.707
							f2	1, 16	38.017	** < 0.001**	0.704
	Time × hemisphere	0.245	8, 9	3.473	**0.041**	0.755	FA^‡^	1.5, 23.8	0.680	0.474	0.041
							MD	2, 32	1.549	0.228	0.088
							AD	2, 32	1.040	0.365	0.061
							f2	2, 32	0.582	0.565	0.035
Adjusted	Time	0.163	8, 7	4.485	**0.031**	0.837	FA	2, 28	3.708	0.037	0.209
							MD	2, 28	2.555	0.096	0.154
							AD	2, 28	4.294	0.024	0.235
							f2	2, 28	0.580	0.566	0.040
	Hemisphere	0.426	4, 11	3.709	**0.038**	0.574	FA	1, 14	0.058	0.813	0.004
							MD	1, 14	13.039	**0.003**	0.482
							AD	1, 14	12.035	**0.004**	0.462
							f2	1, 14	6.136	0.027	0.305
	Affected side	0.603	4, 11	1.814	0.196	0.397					
	Time since onset	0.825	4, 11	0.585	0.680	0.175					
	Time × hemisphere	0.269	8, 7	2.380	0.135	0.731					
	Time × affected side	0.387	8, 7	1.389	0.339	0.613					
	Time × time since onset	0.177	8, 7	4.057	**0.041**	0.823	FA	2, 28	2.396	0.109	0.146
							MD	2, 28	1.945	0.162	0.122
							AD	2, 28	2.886	0.073	0.171
							f2	2, 28	0.694	0.508	0.047
	Hemisphere × affected side	0.864	4, 11	0.431	0.783	0.136					
	Hemisphere × time since onset	0.703	4, 11	1.161	0.379	0.297					
	Time × hemisphere × affected side	0.200	8, 7	3.502	0.058	0.800					
	Time × hemisphere × time since onset	0.316	8, 7	1.894	0.207	0.684					

*Order: hypothesis df, error df; ^†^Bonferroni-corrected significance level *p* < 0.0125 for *n* = 4 marked in bold; ^‡^Greenhouse‒Geisser corrected univariate statistics with *ε* = 0.743 due to sphericity assumption violation, *p* = 0.041, Mauchly’s test of sphericity.

*AD* axial diffusivity, *ANOVA* analysis of variance, *df* degrees of freedom, *f2* secondary partial volume fraction, *FA* fractional anisotropy, *MANOVA* multivariate ANOVA, *MD* mean diffusivity.

In a sensitivity analysis adjusted for the time since onset and the affected side (i.e., affected optic nerve), significant multivariate effects of time, hemisphere and interaction time × time since onset were detected. The post hoc tests for time × time since onset as well as the main effect of time were non-significant (however, time was significant for FA and AD at the uncorrected level, see Table 5), whereas the effect of hemisphere remained significant for MD and AD (Table 5 and Fig. 1).

Finally, the longitudinal MANOVA stratified according to outcomes (Hypothesis 2b) yielded significant multivariate effects of abnormal outcome, hemisphere and interaction time × hemisphere. Whereas the interaction was not confirmed in the univariate post hoc tests, the main effect of abnormal outcome was significant for FA, with lower FA observed in patients with incomplete recovery (Fig. 2). The effect of hemisphere was significant for MD, AD, and f2 (Table 6).

**Table 6:** MANOVA and ANOVA for longitudinal assessment in patients, stratified by outcome.

MANOVA							ANOVA				
Model	Factor	Wilk’s λ	df*	*F*	*p*	Partial *η*^*2*^	Variable	df*	*F*	*p*^†^	Partial *η*^*2*^
Stratified by outcome	Abnormal outcome	0.304	4, 12	6.883	**0.004**	0.696	FA	1, 15	8.058	**0.012**	0.349
							MD	1, 15	0.424	0.525	0.027
							AD	1, 15	3.037	0.102	0.168
							f2	1, 15	1.566	0.230	0.095
	Time	0.641	8, 8	0.560	0.785	0.359					
	Hemisphere	0.263	4, 12	8.396	**0.002**	0.737	FA	1, 15	3.911	0.067	0.207
							MD	1, 15	11.436	**0.004**	0.433
							AD	1, 15	28.808	** < 0.001**	0.658
							f2	1, 15	27.090	** < 0.001**	0.644
	Abnormal outcome × time	0.636	8, 8	0.571	0.777	0.364					
	Abnormal outcome × hemisphere	0.856	4, 12	0.503	0.734	0.144					
	Time × hemisphere	0.153	8, 8	5.529	**0.013**	0.847	FA	2, 30	1.763	0.189	0.105
							MD	2, 30	1.041	0.365	0.065
							AD	2, 30	1.270	0.295	0.078
							f2	2, 30	0.855	0.435	0.054
	Abnormal outcome × time × hemisphere	0.253	8, 8	2.947	0.074	0.747					

*Order: hypothesis df, error df; ^†^Bonferroni-corrected significance level *p* < 0.0125 for *n* = 4 marked in bold.

*AD* axial diffusivity, *ANOVA* analysis of variance, *df* degrees of freedom, *f2* secondary partial volume fraction, *FA* fractional anisotropy, *MANOVA* multivariate ANOVA, *MD* mean diffusivity.

Most of these results were reproduced in the sensitivity analysis using corresponding models, in which FA was replaced by f1 (Supplementary Tables 4 and 5). However, no significant univariate effect of abnormal outcomes was detected in the stratified model (although f1 was significant at the uncorrected level; Supplementary Table 5).

### Correlations

There was no significant correlation between ophthalmological assessments (BCVA, Pelli‒Robson score, RNFL, AS, VS, AVD) and DWI parameters at corresponding time points (Hypothesis 3) at the Bonferroni–Holm-corrected *alpha* = 0.0083, see Supplementary Table 6.

However, a significant correlation was observed between baseline AD in the left OR and follow-up VS of the AE (Hypothesis 4, Spearman’s *ρ* =  − 0.616, *p* = 0.006, *n* = 18). At the uncorrected level, higher baseline FA additionally tended to be associated with higher BCVA and Pelli–Robson at M6 (see Table 7).

**Table 7:** Prediction of ophthalmological parameters at M6 by baseline parameters.

			M6											
			BCVA		Pelli‒Robson		RNFL		AS		VS		AVD	
			ρ*	*p*^†^	ρ*	*p*^†^	ρ*	*p*^†^	ρ*	*p*^†^	ρ*	*p*^†^	ρ*	*p*^†^
M0	*n*		18		15		18		18		18		18	
	FA	L	0.483	0.042	0.304	0.271	0.037	0.884	0.175	0.487	0.064	0.802	0.088	0.729
		R	0.517	0.028	0.567	0.028	0.244	0.330	0.262	0.294	0.055	0.827	0.132	0.601
	MD	L	− 0.204	0.416	− 0.196	0.483	0.039	0.877	− 0.046	0.855	− 0.441	0.067	0.257	0.302
		R	− 0.142	0.575	− 0.397	0.143	0.156	0.538	− 0.082	0.747	− 0.190	0.449	0.154	0.543
	AD	L	0.272	0.276	− 0.122	0.666	0.086	0.733	0.219	0.382	** − 0.616**	**0.006**	0.577	0.012
		R	0.224	0.371	0.043	0.880	0.440	0.068	0.098	0.700	− 0.258	0.301	0.390	0.109
	RD	L	− 0.339	0.169	− 0.233	0.403	0.038	0.880	− 0.176	0.485	− 0.236	0.346	0.072	0.777
		R	− 0.327	0.186	− 0.458	0.086	− 0.106	0.674	− 0.184	0.464	− 0.264	0.290	0.083	0.744
	f1	L	0.432	0.073	0.235	0.400	− 0.020	0.938	0.203	0.418	0.180	0.476	0.036	0.887
		R	0.378	0.122	0.498	0.059	0.166	0.510	0.323	0.191	0.133	0.600	0.127	0.615
	f2	L	− 0.208	0.407	0.138	0.625	0.005	0.984	− 0.363	0.139	− 0.130	0.606	− 0.079	0.757
		R	0.290	0.242	0.081	0.774	− 0.040	0.874	− 0.286	0.250	− 0.091	0.720	− 0.215	0.392

*Spearman rank correlation coefficient; ^†^Bonferroni–Holm-corrected significance level *p* < 0.0083 (*n* = 6). Significant correlations marked in bold.

*AD* axial diffusivity, *AS* arteriolar (oxygen) saturation, *AVD* arterio-venous difference, *BCVA* Best-Corrected Visual Acuity, *DWI* diffusion-weighted imaging, *f2* secondary partial volume fraction, *FA* fractional anisotropy, *L* left, *M0* visit at month 0 (baseline), *M3* visit at month 3, *M6* visit at month 6, *MD* mean diffusivity, *R* right, *RNFL* retinal nerve fiber layer, *VS* venular (oxygen) saturation.

For auxiliary hypotheses 5–7, several significant correlations were observed at the uncorrected alpha = 0.05 (Supplementary Table 7 for the full results), including negative correlation between AVD and BCVA at baseline (*ρ* =  − 0.604, *p* = 0.003, *n* = 22) and a positive correlation between RNFL and BCVA at M3 and M6 (M3: *ρ* = 0.466, *p* = 0.039, *n* = 20; M6: *ρ* = 0.495, *p* = 0.031, *n* = 19). Retinal VS of the AE at M0 significantly predicted BCVA at M6 (*ρ* = 0.609, *p* = 0.007, *n* = 18), and a similar trend was observed for AVD (*ρ* =  − 0.410, *p* = 0.091, *n* = 18). Both the VS and AVD of the AE significantly predicted the RNFL at M6 (VS: *ρ* = 0.571, *p* = 0.013; AVD: *ρ* =  − 0.747, *p* =  < 0.001, *n* = 18). Finally, no significant correlation between DWI parameters and lesion load (including the lesion volume fraction of the OR) was observed at any time point at *p* < 0.05 uncorrected (Hypothesis 8); see Supplementary Table 8.

### ROC analysis

**Fig. 3:**
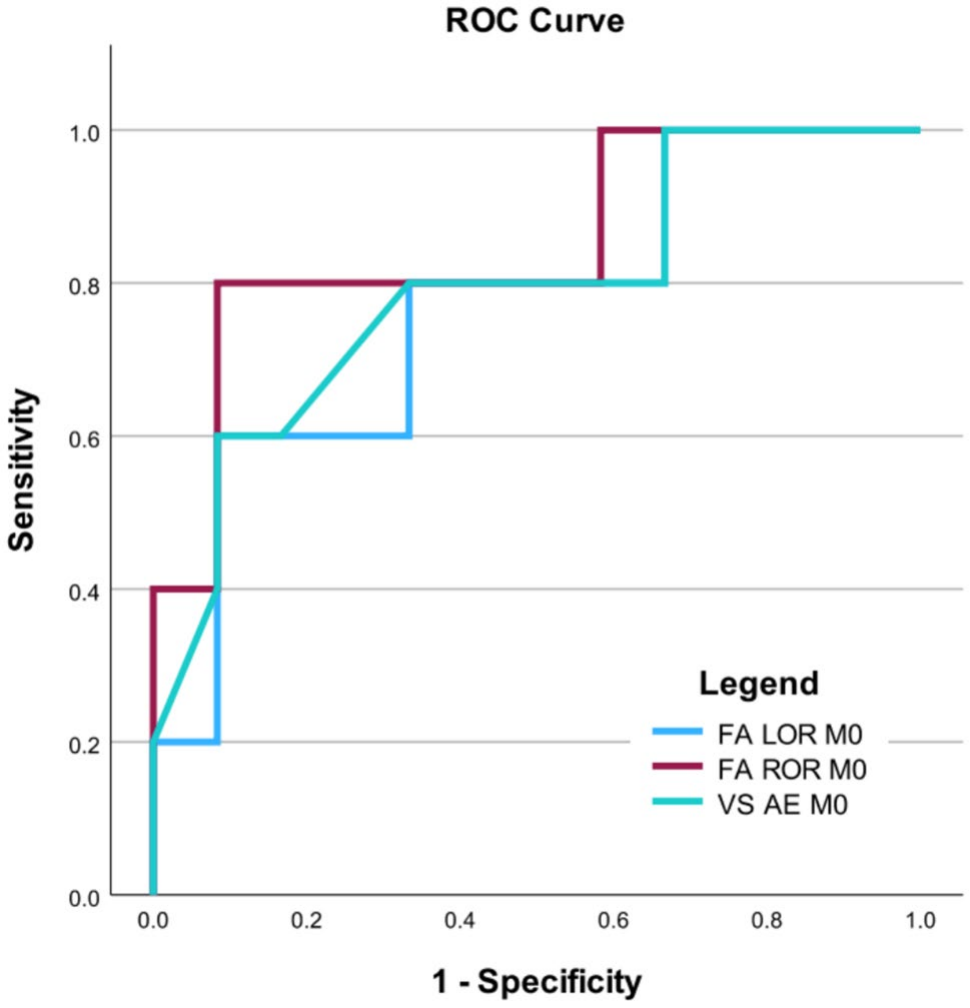
Receiver operating characteristic (ROC) curve for prediction of incomplete recovery. The ROC curve illustrates sensitivity vs. 1 − specificity for fractional anisotropy (FA) of the left and right optic radiation (LOR and ROR, respectively) and retinal venular saturation (VS) of the affected eye (AE) at baseline (M0) for predicting abnormal outcomes at month 6, i.e., best-corrected visual acuity (BCVA) < 20/20 and/or Pelli‒Robson score < 1.65 log of the AE.

The post hoc ROC analysis was performed for FA of the left and right ORs, and for VS of the AE. We observed above-chance AUCs for two of the three tested predictors (FA of the right OR: AUC = 0.850, *p* = 0.004; FA of the left OR: AUC = 0.767, *p* = 0.057; and VS of the AE: AUC = 0.792, *p* = 0.032, *n* = 17, asymptotic two-tailed significance for null hypothesis AUC = 0.5, Fig. 3). No significant paired-sample differences were observed among the three ROC curves.

## Discussion

While we observed significantly greater f2 in patients than in controls in the sex-matched healthy control subgroup, no parameters derived from the standard DTI model (FA, MD, AD) differed between the patient and HC groups (FA, however, differed at the uncorrected level in the matched sample). Similarly, previous studies reported no or only a minimal difference in FA in the OR in patients with acute or early ON^42–45^. This finding indicates that acute ON is not associated with any substantial changes in classical DTI parameters in the OR, although decreases in FA have been reported in normal-appearing white matter (NAWM) of the OR in patients 1–4 years after ON^46–49^, or in MS regardless of ON history^16,50,51^.

To our knowledge, there has been no prior study assessing DWI parameters in the OR after ON using the multicompartment ball-and-stick diffusion model. In this study, f1 was strongly correlated with FA, suggesting that f1 conveys little to no additional information on top of FA. In fact, the additionally evaluated group differences in f1 were observed only at the uncorrected level, i.e., similar to FA. In contrast, marked group differences were found for f2, which represents the secondary fiber population within each voxel, i.e., crossing, kissing, or fanning fibers^24,52^. We speculate that the increased proportion of crossing fibers in patients with ON could result from a relative loss of the primary fiber population oriented parallel to the OR, which could precede the decrease in overall FA observed later in the disease course^47,49^. The finding of sex-related differences in f2 in the adjusted model suggests, however, preexisting microstructural sexual dimorphism in the OR similar to previously reported sex-related differences in classical DTI parameters^53^. Moreover, considerable lateralization was detected for all DWI parameters and across both groups in this study. Although a systematic methodological bias (e.g., due to asymmetric tractography) cannot be ruled out, corresponding left‒right differences in the OR have been reported previously^53,54^. Taken together, the observed lateralization and sexual dimorphism hinder the interpretability of the group differences in f2 and indicate that its use as a disease biomarker would be challenging.

With respect to classical DTI parameters, several previous studies have shown a gradual decline in the FA of the OR following ON but no changes in AD or MD^43,45^. Our data revealed significant multivariate changes in DWI parameters over time in a sensitivity analysis with an adjusted model, with post hoc *F* tests indicating possible effects for FA and AD at the uncorrected level. This was observed only when adjusting for the time since ON onset at the M0 visit (also with significant multivariate interaction time × time since onset), suggesting that changes in DWI parameters are likely to be nonlinear. Although Tur et al.^45^ reported no evidence of nonlinear effects, future studies should consider nonlinear trajectory and/or time since symptom onset as a covariate when assessing DWI parameters after ON.

Importantly, our model stratified according to outcomes revealed significantly lower FA in patients who had incomplete recovery after 6 months but detected no interaction of abnormal outcome × time. This finding indicates that patients who remain clinically affected 6 months after ON have overall lower FA, but their rate of FA decline (or increase) does not substantially differ from that of patients with a complete recovery, suggesting that changes due to inflammatory damage and remyelination propagating along the visual pathway into the OR may have similar kinetics in both groups. However, a significant multivariate interaction time × hemisphere and a statistically non-significant yet potentially relevant trend for the interaction of abnormal outcome × time × hemisphere were observed (*p* = 0.074, Table 6). While a possible significant interaction of abnormal outcome × time ﻿cannot be ruled out in a larger sample, the lack thereof in the current analysis may potentially arise as a consequence of lateralization effects.

Contrary to our expectations, neither visual function outcomes nor the RNFL were significantly correlated with DWI at the respective time points. However, the lack of a significant correlation is generally in line with the findings of previous studies in comparable cohorts. Several studies in patients after ON detected no significant correlations between DWI parameters in the OR and visual acuity or the RNFL^42,44,49–51^. In another study, changes in the RNFL correlated with annualized changes in AD but not in FA^43^. However, studies in larger samples, including patients with MS regardless of ON history, revealed significant correlations of FA with RNFL^16,48,55^ and visual acuity when controlling for DWI parameters outside the OR^16^. Hence, larger sample sizes and/or longer follow-up periods are warranted to confirm the relationships among DWI parameters, visual acuity and RNFL thinning after ON.

As in the case of cross-sectional correlations, we observed no significant relationship between baseline DWI and follow-up visual function assessment or RNFL at the corrected significance level. However, because of the mutual correlation of multiple independent variables tested here, the application of Bonferroni correction might be too conservative. Instead, a biologically plausible relationship can be inferred on the basis of a consistent pattern rather than statistical significance^16^. In fact, we detected several such consistent associations between visual function outcomes (both BCVA and contrast sensitivity) and baseline FA at the uncorrected level. Owing to the low number of abnormal findings in BCVA and contrast sensitivity assessments at M6, the performance of the predictors was evaluated in an additional ROC analysis using a dichotomized aggregate visual function outcome. Here, FA provided good discrimination (AUCs of 0.850 and 0.767 for the right and left ORs, respectively) between patients with complete and incomplete recovery. To our knowledge, no DWI-based predictors of visual outcomes after acute ON have been previously identified in the OR^45^. Although a recent study revealed that AD and RD at 1 or 3 months after ON onset were predictive of RNFL at the subsequent 6-month and 12-month follow-up visits, no prediction of visual acuity at the 6-month or 12-month visit was detected^18^. Hence, independent validation of FA as a predictor of visual outcomes in a larger dataset is still needed.

Despite the lack of significant cross-sectional correlations, we detected a significant negative association between baseline AD in the left OR and follow-up retinal VS in the affected eye (corrected for the number of predicted variables). Our previous study in a larger cohort of patients with acute ON revealed a decrease in AVD along with a trend toward increased VS in the affected and fellow eyes after a 6-month follow-up, which was suggested to reflect retinal atrophy^13^. The potential mechanism linking DWI and oximetry thus might involve neurodegeneration as decreases in AD have also been associated with axonal loss^56^. The histopathological interpretation of AD remains, however, challenging when mixed pathology is expected as in the case of MS^56^, rendering the proposed explanation to a large degree speculative. The consistent correlations at M0 and M6 nevertheless indicate that the early microstructural features of the OR that are predictive of follow-up AVD are preserved throughout the 6-month follow-up period.

Our auxiliary analyses yielded several additional interrelated findings. First, the baseline AVD was correlated with baseline visual impairment. Second, baseline AVD and VS predicted the follow-up RNFL, whereas VS also predicted follow-up visual acuity (and a trend was detected for AVD). Finally, the BCVA and RNFL at M6 were also correlated with each other. Taken together, these correlations suggest that increased oxygen metabolism at baseline (as reflected by lower VS and higher AVD)^13,31^ corresponds to the extent of inflammation and the resulting acute and residual vision impairment and retinal atrophy. These are novel results, as the predictive utility of retinal oximetry for functional outcomes or RNFL thinning after ON has not been assessed before. The AUC for VS was comparable to the AUC for FA, suggesting that both baseline parameters are promising potential biomarkers of visual function recovery. Although, to our knowledge, neither retinal oximetry nor DTI parameters are currently employed in diagnostic or treatment algorithms in ON or in MS and they are still considered experimental^57^, early identification of patients with ON at risk of permanent visual impairment might enable personalized ON treatment, such as preference of plasma exchange over methylprednisolone or preference of certain disease-modifying regimes. Future studies should assess the combined performance of FA and VS (here, logistic regression analysis was not feasible because of an insufficient sample size).

Several limitations must be acknowledged. First, the relatively small sample size could have negatively affected the power of our tests. The sample size was affected by a large dropout rate due to refusal to undergo MRI, which was possibly related to ongoing COVID-19 pandemics at the time of recruitment. Nevertheless, previous studies in similar cohorts with acute ON had comparable sample sizes^45^, and our significant results indicate a sufficient sample size for large-sized effects. A validation in a larger independent cohort is, however, warranted. Second, in this study, the whole OR of each participant was assessed. While we did not observe any correlation with lesion load, local effects in the OR due to intersecting T2 lesions cannot be ruled out, and a future assessment of NAWM is warranted. As in each DWI study, selection of the fiber tract of interest is challenging. Here, a robust and reproducible method using a widely available toolbox (XTRACT) was employed to maximize the replicability of our results. Furthermore, distinguishing among optic nerve  likely affecting fibers of the right, the left or both ORs (i.e., affecting one or both hemifields) was not possible. Future studies should thus consider the potential relationship between the lesion/symptom pattern and the side of the OR. Finally, we deliberately ignored the effect of treatment. In our cohort, each patient received systemic corticoids at M0; hence, our results cannot be generalized to untreated subjects. Disease-modifying treatment was initiated in some patients at the discretion of the treating physician as per local guidelines. Owing to unbalanced distribution, variable timing and heterogeneity of initiated treatments, an analysis of treated vs. untreated patients was not deemed informative. Observational studies in larger cohorts and possibly interventional studies are warranted to establish the effect of early treatment initiation in ON and to test whether the results can be generalized to any disease-modifying treatment.

In summary, while optic neuritis (ON) was not associated with microstructural changes in the optic radiations (ORs) that would consistently affect all patients, incomplete recovery of visual functions at the six-month follow-up was associated with lower baseline fractional anisotropy (FA) and increased retinal oxygen metabolism. Retinal oximetry additionally predicted the subsequent development of retinal nerve fiber layer (RNFL) atrophy. Our results suggest the possible utility of DWI and oximetry for the stratification of patients with ON in future studies investigating novel therapeutic interventions.

## Supplementary Information

Supplementary Information.

## Data Availability

The datasets generated during and/or analyzed during the current study are available from the corresponding author upon reasonable request.
