# Supplementary material for: Diffusion-weighted imaging and retinal oximetry as potential biomarkers of visual outcomes after optic neuritis
Source: Sci Rep. 2025 Oct 10;15:35445. doi: 10.1038/s41598-025-19331-w (PMC12514231; doi:10.1038/s41598-025-19331-w)
Supplement: Supplementary file 1 — Supplementary Information. [file 41598_2025_19331_MOESM1_ESM.pdf]

Supplementary Material

Diffusion-weighted imaging and retinal oximetry as potential biomarkers of visual outcomes after optic neuritis

Pavel Hok[*,1,2,3,11], Jan Valošek[1,4,5], Tereza Králová[1,6], František Odstrčil[7], Martina Sapieta[8], Michal Král[1,6], Kruznev S. Nijhar[1,6], Anna Arkhipova[1,6], Monika Jasenská[1,9], Jan Mareš[1,6], Martin Šín[8,10]

[1]Department of Neurology, Faculty of Medicine and Dentistry, Palacký University Olomouc, Olomouc, Czechia;
[2]Department of Neurology, University Medicine Greifswald, Greifswald, Germany;
[3]Functional Imaging Unit, Institute of Diagnostic Radiology and Neuroradiology, University Medicine Greifswald, Greifswald, Germany;
[4]Department of Neurosurgery, Faculty of Medicine and Dentistry, Palacký University Olomouc, Olomouc, Czechia;
[5]NeuroPoly Lab, Institute of Biomedical Engineering, Polytechnique Montreal, Montreal, QC, Canada;
[6]Department of Neurology, University Hospital Olomouc, Olomouc, Czechia;
[7]Department of Radiology, University Hospital Olomouc, Olomouc, Czechia;
[8]Department of Ophthalmology, University Hospital Olomouc, Olomouc, Czechia;
[9]Department of Biomedical Engineering, University Hospital Olomouc, Olomouc, Czechia;
[10]Department of Ophthalmology, Military University Hospital Prague, Prague, Czechia;
[11]Behavioural and Social Neuroscience, CEITEC – Central European Institute of Technology, Masaryk University, Brno, Czechia.

[*)]Corresponding author: Pavel Hok

Present address: CEITEC – Central European Institute of Technology
Masaryk University
Kamenice 753/5
62500 Brno, Czechia

E-mail: pavel.hok@ceitec.muni.cz

Supplementary Methods

1. Doubly Multivariate Analysis of Variance (MANOVA) procedure

Each MANOVA was interpreted according to the following procedure[1] using SPSS Statistics 30 (IBM, Armonk, NY, USA, https://www.ibm.com/analytics/spss-statistics-software): First, multivariate Wilk's lambda test was performed. For each significant multivariate test ($p < 0.05$), corresponding post hoc univariate statistics (i.e., F tests resulting from a series of one-way analyses of variance [ANOVA]) were assessed for each dependent variable at Bonferroni-corrected $p < 0.0125$. In the case of a significant multivariate interaction with a significant corresponding post hoc univariate interaction, simple mean effects were evaluated.

2. Sensitivity Analyses

For Hypothesis 1, the comparison between patients and healthy controls (HCs) at baseline (M0) was repeated in two sensitivity analyses. In one analysis, MANOVA was adjusted by adding sex and age as additional covariates. In the second analysis, an unadjusted model was used to compare patients to a subset of matched HCs selected via the SPSS matching algorithm controlling for sex, age, ocular dominance, and handedness.

For Hypothesis 2a (longitudinal analysis without stratification according to abnormal outcome), a single sensitivity analysis was carried out by adding the time since onset at M0 and the affected side (left or right) as additional covariates to the MANOVA model.

3. Auxiliary Correlations

For auxiliary Hypotheses 5-7, the associations between visual function (BCVA and Pelli–Robson of the affected side) and ipsilateral retinal parameters (RNFL, AS, VS, and AVD) were evaluated at corresponding time points (Hypothesis 5), as were the associations between baseline retinal parameters and follow-up visual function (Hypothesis 6) or RNFL (Hypothesis 7). Finally, the correlation between DWI parameters and lesion load, including the lesion fraction of the OR, was assessed at corresponding time points (hypothesis 8). Correlations for the main hypotheses were considered significant at $p < 0.05$ with additional Bonferroni–Holm correction for the number of independent variables.

4. References

1. Pituch, K. A. & Stevens, J. P. *Applied Multivariate Statistics for the Social Sciences: Analyses with SAS and IBM's SPSS, Sixth Edition*. (Routledge, 2015).

Supplementary figures

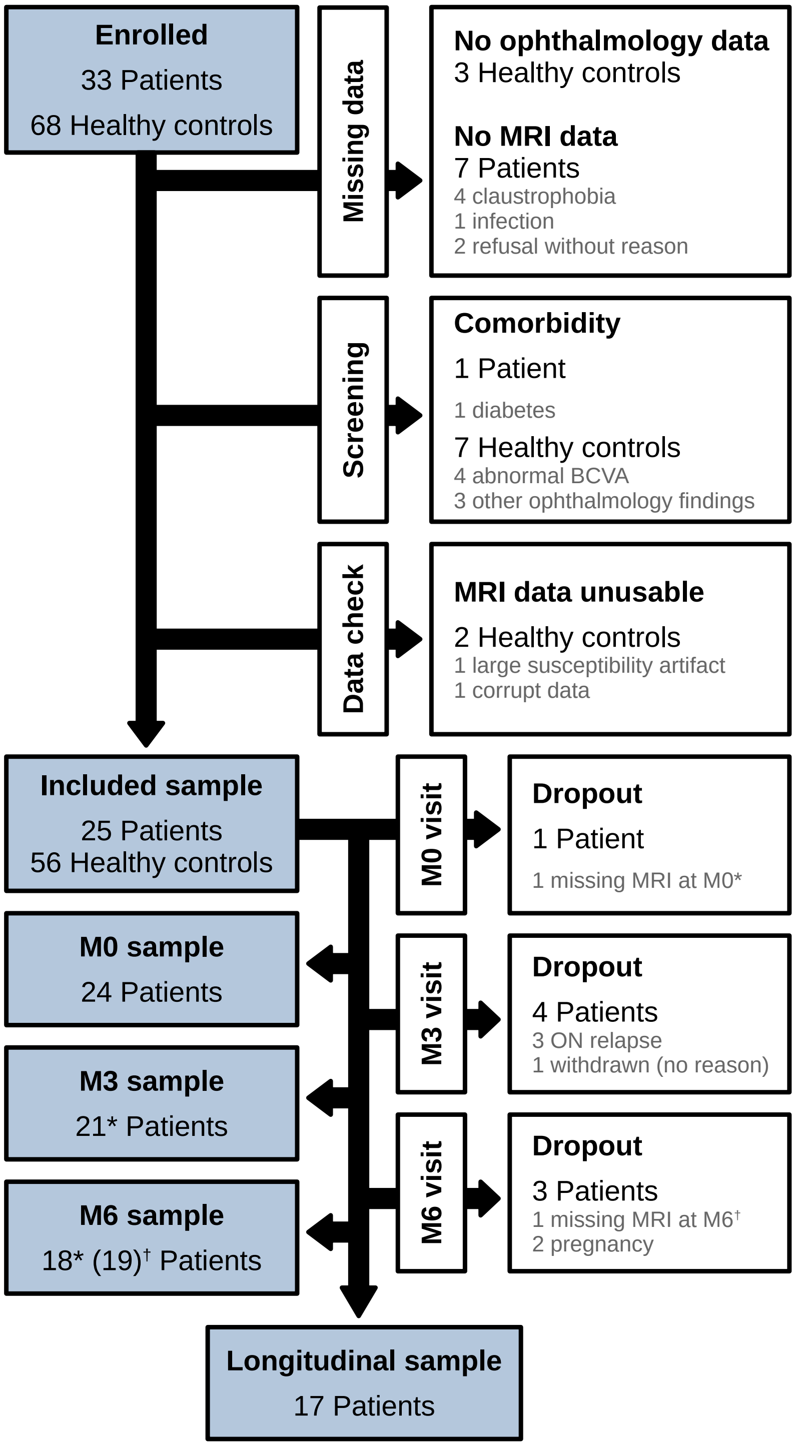

Supplementary Fig. S1. Exclusion chart. Abbreviations: BCVA (best-corrected visual acuity); M0 – month 0; M3 – month 3; M6 – month 6; MRI – magnetic resonance imaging; ON – optic neuritis.
Notes: *) Patient without MRI data at M0 was included in correlation analyses of the M3 and M6 samples; †) Patient without MRI data at M6 was included in auxiliary analyses of M6 sample.

Supplementary Tables

Supplementary Table 1. Summary of outcome measures, regressors and statistical tests

**Number**	**Hypothesis**	**Outcome measures (dependent variables)**	**Explanatory variables**	**Confounders**	**Statistical Test**
**1**	Group differences in DWI parameters	FA, MD, AD, RD[a], f1[a], f2 (LOR/ROR) at M0	group, hemisphere	none	MANOVA
				age, sex	
**2**	Change in DWI parameters over time	FA, MD, AD, RD[a], f1[a], f2 (LOR/ROR) at M0, M3, and M6	time, hemisphere	none	MANOVA
				affected side, time since onset	
			time, hemisphere, abnormal outcome	none	MANOVA
**3**	Correlation with clinical parameters and retinal imaging	BCVA, Pelli–Robson score, RNFL AS, VS, AVD of the AE at M0, M3, and M6	FA, MD, AD, RD, f1, f2 (LOR/ROR) at M0, M3, and M6	none	Spearman rank correlation coefficient
**4**	Prediction of clinical parameters and retinal imaging at M6	BCVA, Pelli–Robson score, RNFL AS, VS, AVD of the AE at M6	FA, MD, AD, RD, f1, f2 (LOR/ROR) at M0	none	Spearman rank correlation coefficient
			**Auxiliary hypotheses**		
**5**	Correlation between clinical parameters and retinal imaging	BCVA, Pelli–Robson score of the AE at M0, M3, and M6	RNFL AS, VS, AVD of the AE at M0, M3, and M6	none	Spearman rank correlation coefficient
**6**	Prediction of clinical parameters at M6	BCVA, Pelli–Robson score of the AE at M6	RNFL AS, VS, AVD of the AE at M0	none	Spearman rank correlation coefficient
**7**	Prediction of retinal atrophy at M6	RNFL of the AE at M6	AS, VS, AVD of the AE at M0	none	Spearman rank correlation coefficient
**8**	Correlation with lesion load	FA, MD, AD, RD, f1, f2 (LOR/ROR) at M0, M3, and M6	LL at M0, M3, and M6	none	Spearman rank correlation coefficient

Notes: a)Variables excluded from the main analysis due to multicollinearity.

Abbreviations: AD – axial diffusivity; AE – affected eye; AS – arteriolar (oxygen) saturation; AVD – arteriovenous difference; BCVA – Best-Corrected Visual Acuity; f1 – primary partial volume fraction; f2 – secondary partial volume fraction; FA – fractional anisotropy; LL – lesion load; LOR – left optic radiation; M0 – month 0 (baseline); M3 – month 3; M6 – month 6; MANOVA – multivariate analysis of variance; MD – mean diffusivity; RD – radial diffusivity; RNFL – retinal nerve fiber layer; ROR – right optic radiation; VS – venular (oxygen) saturation.

Supplementary Table 2. Summary DWI measures

		Patients with optic neuritis						HCs	
		M0[a]		M3[b]		M6[b]		M0	
n		24		17		17		56	
		Mean	SD	Mean	SD	Mean	SD	Mean	SD
FA	L	0.57	0.03	0.57	0.03	0.57	0.03	0.58	0.02
	R	0.56	0.03	0.56	0.03	0.56	0.03	0.57	0.02
MD [$\times 10^{-3}$ mm^2/s]	L	0.81	0.03	0.81	0.03	0.80	0.03	0.80	0.03
	R	0.79	0.03	0.79	0.02	0.79	0.03	0.78	0.03
AD [$\times 10^{-3}$ mm^2/s]	L	1.40	0.05	1.39	0.05	1.38	0.05	1.40	0.05
	R	1.34	0.05	1.34	0.04	1.34	0.04	1.35	0.05
RD [$\times 10^{-3}$ mm^2/s]	L	0.51	0.03	0.51	0.03	0.51	0.03	0.50	0.03
	R	0.51	0.03	0.51	0.03	0.51	0.03	0.50	0.03
f1	L	0.45	0.03	0.44	0.03	0.44	0.03	0.46	0.02
	R	0.43	0.03	0.43	0.02	0.44	0.02	0.44	0.02
f2	L	0.13	0.01	0.13	0.01	0.13	0.01	0.12	0.02
	R	0.15	0.02	0.15	0.01	0.14	0.01	0.14	0.02

Notes: [a)]all included subjects; [b)]Patients with optic neuritis included in longitudinal multivariate analysis of variance.

Abbreviations: AD – axial diffusivity; DWI – diffusion-weighted imaging; f1 – primary partial volume fraction; f2 – secondary partial volume fraction; FA – fractional anisotropy; HCs – healthy controls; L – left; MD – mean diffusivity; R – right; RD – radial diffusivity; SD – standard deviation.

Supplementary Table 3. MANOVA and post hoc ANOVA for group comparison at baseline (analysis with f1)

	MANOVA						ANOVA				
**Model**	**factor**	**Wilk's λ**	**df[a]**	***F***	***p***	**Partial η^2**	**Variable**	**df[a]**	***F***	***p*[b]**	**Partial η^2**
**Unadjusted 24 patients vs. 56 HCs**	group	0.841	4, 75	3.554	**0.010**	0.159	MD	1, 78	0.639	0.427	0.008
							AD	1, 78	0.148	0.702	0.002
							f1	1, 78	4.147	0.045	0.050
							f2	1, 78	8.799	**0.004**	0.101
	hemisphere	0.325	4, 75	38.857	**<0.001**	0.675	MD	1, 78	49.453	**<0.001**	0.388
							AD	1, 78	138.484	**<0.001**	0.640
							f1	1, 78	29.033	**<0.001**	0.271
							f2	1, 78	81.230	**<0.001**	0.510
	group×hemisphere	0.949	4, 75	1.001	0.412	0.051					
**Adjusted 24 patients vs. 56 HCs**	group	0.904	4, 72	1.920	0.116	0.096					
	hemisphere	0.917	4, 72	1.632	0.175	0.083					
	age	0.934	4, 72	1.266	0.291	0.066					
	sex	0.790	4, 72	4.787	**0.002**	0.210	MD	1, 75	0.415	0.521	0.006
							AD	1, 75	4.185	0.044	0.053
							f1	1, 75	2.688	0.105	0.035
							f2	1, 75	16.643	**<0.001**	0.182
	group×sex	0.965	4, 72	0.654	0.626	0.035					
	group×hemisphere	0.972	4, 72	0.514	0.725	0.028					
	hemisphere×age	0.976	4, 72	0.449	0.772	0.024					
	hemisphere×sex	0.992	4, 72	0.147	0.964	0.008					
	group× hemisphere×sex	0.992	4, 72	0.140	0.967	0.008					
**Unadjusted 24 patients vs. 24 HCs**	group	0.759	4, 43	3.412	**0.016**	0.241	MD	1, 46	0.533	0.469	0.011
							AD	1, 46	1.266	0.266	0.027
							f1	1, 46	6.437	0.015	0.123
							f2	1, 46	7.795	**0.008**	0.145
	hemisphere	0.305	4, 43	24.542	**<0.001**	0.695	MD	1, 46	41.767	**<0.001**	0.476
							AD	1, 46	93.242	**<0.001**	0.670
							f1	1, 46	20.792	**<0.001**	0.311
							f2	1, 46	48.213	**<0.001**	0.512
	group×hemisphere	0.934	4, 43	0.757	0.559	0.066					

Notes: [a)]Order: hypothesis df, error df; [b)]Bonferroni-corrected significance level $p < 0.0125$ for $n = 4$ marked in **bold**.

Abbreviations: AD – axial diffusivity; ANOVA – analysis of variance; df – degrees of freedom; f1 – primary partial volume fraction; f2 – secondary partial volume fraction; FA – fractional anisotropy; HCs – healthy controls; MANOVA – multivariate ANOVA; MD – mean diffusivity.

Supplementary Table 4. MANOVA and post hoc ANOVA for longitudinal assessment in patients, irrespective of outcome (analysis with f1)

	MANOVA						ANOVA				
**Model**	**factor**	**Wilk's λ**	**df[a]**	***F***	***p***	**Partial η^2**	**Variable**	**df[a]**	***F***	***p*[b]**	**Partial η^2**
Unadjusted	time	0.519	8, 9	1.041	0.472	0.481					
	hemisphere	0.209	4, 13	12.279	**<0.001**	0.791	MD	1, 16	15.308	**0.001**	0.489
							AD	1, 16	38.641	**<0.001**	0.707
							f1	1, 16	3.977	0.063	0.199
							f2	1, 16	38.017	**<0.001**	0.704
	time×hemisphere	0.354	8, 9	2.056	0.152	0.646					
Adjusted	time	0.190	8, 7	3.729	**0.050**	0.810	MD	2, 28	2.555	0.096	0.154
							AD	2, 28	4.294	0.024	0.235
							f1	2, 28	1.636	0.213	0.105
							f2	2, 28	0.580	0.566	0.040
	hemisphere	0.445	4, 11	3.424	**0.047**	0.555	MD	1, 14	13.039	**0.003**	0.482
							AD	1, 14	12.035	**0.004**	0.462
							f1	1, 14	0.005	0.943	0.000
							f2	1, 14	6.136	0.027	0.305
	affected side	0.604	4, 11	1.804	0.198	0.396					
	time since onset	0.754	4, 11	0.897	0.498	0.246					
	time×hemisphere	0.412	8, 7	1.249	0.391	0.588					
	time×affected side	0.189	8, 7	3.763	**0.049**	0.811b	MD	2, 28	1.438	0.254	0.093
							AD	2, 28	2.448	0.105	0.149
							f1	2, 28	1.435	0.255	0.093
							f2	2, 28	0.046	0.955	0.003
	time× time since onset	0.213	8, 7	3.231	0.070	0.787					
	hemisphere× affected side	0.882	4, 11	0.367	0.827	0.118					
	hemisphere× time since onset	0.679	4, 11	1.303	0.328	0.321					
	time×hemisphere× affected side	0.347	8, 7	1.645	0.263	0.653					
	time×hemisphere× time since onset	0.442	8, 7	1.103	0.455	0.558					

Notes: [a)]Order: hypothesis df, error df; [b)]Bonferroni-corrected significance level $p < 0.0125$ for $n = 4$ marked in **bold**.

Abbreviations: AD – axial diffusivity; ANOVA – analysis of variance; df – degrees of freedom; f2 – secondary partial volume fraction; FA – fractional anisotropy; MANOVA – multivariate ANOVA; MD – mean diffusivity.

Supplementary Table 5. MANOVA and ANOVA for longitudinal assessment in patients, stratified by outcome (analysis with f1)

		MANOVA					ANOVA				
**Model**	**factor**	**Wilk's λ**	**df[a]**	***F***	***p***	**Partial η^2**	**Variable**	**df[a]**	***F***	***p*[b]**	**Partial η^2**
**Stratified by outcome**	abnormal outcome	0.342	4, 12	5.782	**0.008**	0.658	MD	1, 15	0.424	0.525	0.027
							AD	1, 15	3.037	0.102	0.168
							f1	1, 15	4.650	0.048	0.237
							f2	1, 15	1.566	0.230	0.095
	time	0.518	8, 8	0.929	0.540	0.482					
	hemisphere	0.251	4, 12	8.935	**0.001**	0.749	MD	1, 15	11.436	**0.004**	0.433
							AD	1, 15	28.808	**<0.001**	0.658
							f1	1, 15	2.681	0.122	0.152
							f2	1, 15	27.090	**<0.001**	0.644
	abnormal outcome× time	0.469	8, 8	1.134	0.432	0.531					
	abnormal outcome× hemisphere	0.843	4, 12	0.561	0.696	0.157					
	time×hemisphere	0.157	8, 8	5.388	**0.014**	0.843	MD	2, 30	1.041	0.365	0.065
							AD	2, 30	1.270	0.295	0.078
							f1[c]	2, 30	2.818	0.095	0.158
							f2	2, 30	0.855	0.435	0.054
	abnormal outcome× time×hemisphere	0.235	8, 8	3.259	0.057	0.765					

Notes: [a)]Order: hypothesis df, error df; [b)]Bonferroni-corrected significance level $p < 0.0125$ for $n = 4$ marked in **bold**; [c)]Greenhouse–Geisser corrected univariate statistics.

Abbreviations: AD – axial diffusivity; ANOVA – analysis of variance; df – degrees of freedom; f2 – secondary partial volume fraction; FA – fractional anisotropy; MANOVA – multivariate ANOVA; MD – mean diffusivity.

Supplementary Table 6. Correlations between DWI and ophthalmological parameters at each time point

			BCVA		Pelli–Robson		RNFL		AS		VS		AVD	
	n		24		15		24		22		22		22	
			ρ^a	p^b	ρ^a	p^b	ρ^a	p^b	ρ^a	p^b	ρ^a	p^b	ρ^a	p^b
M0	FA	L	0.057	0.791	0.059	0.833	0.470	*0.020*	0.174	0.440	0.219	0.328	0.089	0.694
		R	0.160	0.454	0.097	0.730	0.152	0.477	0.248	0.265	0.314	0.154	−0.057	0.802
	MD	L	0.155	0.470	0.095	0.735	−0.272	0.198	−0.095	0.675	−0.203	0.366	−0.005	0.982
		R	0.137	0.524	−0.087	0.758	−0.188	0.380	−0.010	0.964	0.027	0.906	−0.253	0.257
	AD	L	0.211	0.323	0.205	0.464	0.038	0.861	0.100	0.659	−0.058	0.798	0.052	0.820
		R	0.279	0.187	0.088	0.754	−0.138	0.519	0.158	0.484	0.095	0.675	−0.252	0.259
	RD	L	0.061	0.778	−0.013	0.964	−0.423	*0.039*	−0.073	0.746	−0.235	0.292	−0.029	0.899
		R	−0.001	0.997	−0.136	0.628	−0.273	0.198	−0.144	0.523	−0.125	0.579	−0.156	0.488
	f1	L	−0.016	0.940	−0.002	0.995	0.464	*0.022*	0.178	0.429	0.208	0.353	0.099	0.663
		R	0.072	0.736	0.031	0.914	0.194	0.365	0.245	0.272	0.228	0.308	0.071	0.754
	f2	L	−0.115	0.591	−0.142	0.613	0.171	0.424	−0.162	0.473	−0.229	0.304	0.216	0.335
		R	0.061	0.779	0.079	0.779	0.396	0.055	−0.226	0.313	0.055	0.808	−0.021	0.926
	n		20		15		20		19		19		19	
M3	FA	L	0.266	0.258	0.328	0.232	0.089	0.709	0.216	0.375	0.410	0.081	−0.304	0.205
		R	0.257	0.273	*0.603*	*0.017*	0.165	0.487	0.006	0.980	−0.019	0.940	−0.021	0.932
	MD	L	0.116	0.626	0.069	0.808	0.151	0.526	0.110	0.653	−0.171	0.484	0.241	0.320
		R	0.121	0.611	−0.300	0.276	0.219	0.353	−0.161	0.511	−0.220	0.366	0.187	0.442
	AD	L	0.400	0.080	0.223	0.425	0.293	0.211	0.115	0.640	0.266	0.271	−0.156	0.524
		R	0.224	0.343	0.204	0.466	0.251	0.286	−0.239	0.325	−0.187	0.443	0.074	0.764
	RD	L	−0.107	0.653	−0.115	0.683	−0.053	0.825	−0.093	0.704	−0.269	0.265	0.230	0.344
		R	−0.021	0.932	*−0.607*	*0.017*	0.034	0.887	−0.116	0.637	−0.169	0.490	0.152	0.534
	f1	L	0.244	0.299	0.378	0.164	0.038	0.875	0.214	0.379	0.360	0.130	−0.279	0.248
		R	0.151	0.525	*0.623*	*0.013*	0.058	0.808	0.006	0.980	0.049	0.841	−0.074	0.764
	f2	L	−0.104	0.661	−0.043	0.880	0.221	0.349	−0.185	0.447	−0.301	0.210	0.199	0.414
		R	0.260	0.268	0.189	0.499	0.153	0.519	−0.037	0.880	−0.057	0.818	−0.027	0.912
	n		18		15		18		18		18		18	
M6	FA	L	0.385	0.114	0.479	0.071	−0.141	0.576	0.172	0.495	0.015	0.954	0.162	0.520
		R	0.271	0.277	0.485	0.067	−0.137	0.587	0.297	0.232	−0.079	0.756	0.175	0.488
	MD	L	−0.182	0.471	−0.065	0.818	−0.174	0.491	−0.058	0.818	−0.355	0.149	0.208	0.408
		R	−0.184	0.464	−0.211	0.451	−0.035	0.890	−0.304	0.220	−0.389	0.110	0.188	0.455
	AD	L	0.405	0.095	0.479	0.071	−0.022	0.932	0.058	0.820	−0.235	0.347	0.271	0.277
		R	0.119	0.638	0.249	0.371	−0.121	0.633	0.023	0.926	*−0.553*	*0.017*	*0.515*	*0.029*
	RD	L	−0.150	0.554	−0.306	0.217	0.071	0.778	0.086	0.735	−0.299	0.229	−0.375	0.168
		R	−0.357	0.146	−0.187	0.458	−0.004	0.987	0.173	0.491	−0.139	0.583	−0.302	0.273
	f1	L	0.263	0.291	0.036	0.886	0.213	0.397	−0.119	0.639	0.424	0.080	0.477	0.072
		R	0.249	0.319	0.127	0.616	0.005	0.984	−0.199	0.428	0.201	0.423	*0.564*	*0.028*
	f2	L	−0.125	0.622	0.160	0.570	−0.076	0.763	−0.434	0.072	−0.119	0.637	−0.151	0.550
		R	0.292	0.240	−0.187	0.504	*0.570*	*0.014*	−0.159	0.528	0.181	0.473	−0.458	0.056

Notes: [a)]Spearman rank correlation coefficient; [b)]Significant correlations at the Bonferroni–Holm-corrected significance level $p < 0.0083$ ($n = 6$) marked in **bold**, uncorrected significance ($p < 0.05$) marked in *italics*.

Abbreviations: AD – axial diffusivity; AS – arteriolar (oxygen) saturation; AVD – arteriovenous difference; BCVA – Best-Corrected Visual Acuity; DWI – diffusion-weighted imaging; f2 – secondary partial volume fraction; FA – fractional anisotropy; L – left; M0 – visit at month 0 (baseline); M3 – visit at month 3; M6 – visit at month 6; MD – mean diffusivity; R – right; RNFL – retinal nerve fiber layer; VS – venular (oxygen) saturation.

Supplementary Table 7. Correlations among retinal parameters and visual function

		BCVA			Pelli–Robson			RNFL		
		ρ^a	p	n	ρ^a	p	n	ρ^a	p	n
						**M0**				
**M0**	**RNFL**	0.015	0.945	24	−0.010	0.972	15			
	**AS**	*−0.390*	*0.073*	22	*−0.490*	*0.063*	15			
	**VS**	0.223	0.318	22	−0.229	0.412	15			
	**AVD**	**−0.604**	**0.003**	22	−0.322	0.242	15			
						**M3**				
**M3**	**RNFL**	**0.466**	**0.039**	20	−0.098	0.729	15			
	**AS**	−0.058	0.815	19	−0.158	0.589	14			
	**VS**	−0.313	0.191	19	0.039	0.894	14			
	**AVD**	0.303	0.207	19	−0.156	0.594	14			
						**M6**				
**M6**	**RNFL**	**0.495**	**0.031**	19	0.032	0.906	16			
	**AS**	−0.006	0.980	19	−0.217	0.420	16			
	**VS**	−0.314	0.190	19	−0.372	0.156	16			
	**AVD**	0.190	0.437	19	0.166	0.538	16			
						**M6 (prediction)**				
**M0**	**RNFL**	0.273	0.257	19	0.041	0.881	16	0.047	0.848	19
	**AS**	0.179	0.477	18	0.239	0.391	15	−0.074	0.769	18
	**VS**	**0.609**	**0.007**	18	0.155	0.582	15	**0.571**	**0.013**	18
	**AVD**	*−0.410*	*0.091*	18	0.109	0.698	15	**−0.747**	**<0.001**	18

Notes: [a)]Spearman rank correlation coefficient; [b)]Uncorrected statistics. Significant correlations ($p < 0.05$) marked in bold, trends ($p < 0.10$) marked in italics.

Abbreviations: AD – axial diffusivity; AS – arteriolar (oxygen) saturation; AVD – arteriovenous difference; BCVA – Best-Corrected Visual Acuity; DWI – diffusion-weighted imaging; f2 – secondary partial volume fraction; FA – fractional anisotropy; L – left; M0 – visit at month 0 (baseline); M3 – visit at month 3; M6 – visit at month 6; MD – mean diffusivity; N/A – not applicable; PR – Pelli–Robson score; R – right; RNFL – retinal nerve fiber layer; VS – venular (oxygen) saturation.

Supplementary Table 8. Correlations between DWI parameters and LL

			Whole-brain LL		OR lesion volume fraction	
			**M0 (n = 24)**			
			ρ^a	p^b	ρ^a	p^b
M0	FA	L	−0.086	0.689	−0.155	0.471
		R	−0.113	0.600	−0.031	0.886
	MD	L	0.241	0.258	0.235	0.270
		R	0.120	0.576	0.000	0.998
	AD	L	0.183	0.392	0.189	0.378
		R	0.067	0.756	−0.130	0.546
	RD	L	0.240	0.259	0.212	0.319
		R	0.122	0.569	0.045	0.835
	f1	L	−0.046	0.832	−0.163	0.446
		R	−0.154	0.471	−0.015	0.943
	f2	L	−0.200	0.349	−0.189	0.377
		R	−0.037	0.864	−0.004	0.987
			**M3 (n = 21)**			
M3	FA	L	−0.008	0.973	−0.354	0.115
		R	−0.044	0.849	−0.258	0.258
	MD	L	0.357	0.113	0.378	0.091
		R	0.060	0.797	0.000	1.000
	AD	L	0.248	0.279	−0.070	0.762
		R	0.034	0.884	−0.222	0.334
	RD	L	0.240	0.294	0.382	0.087
		R	0.048	0.836	0.259	0.258
	f1	L	−0.030	0.898	−0.354	0.115
		R	−0.055	0.814	−0.295	0.194
	f2	L	−0.269	0.239	−0.115	0.621
		R	0.081	0.729	0.369	0.099
			**M6 (n = 18)**			
M6	FA	L	0.018	0.945	−0.189	0.453
		R	0.020	0.938	N/A[c]	
	MD	L	0.447	0.063	0.272	0.276
		R	0.160	0.526	N/A[c]	
	AD	L	0.277	0.266	−0.017	0.947
		R	0.127	0.616	N/A[c]	
	RD	L	0.159	0.528	0.257	0.303
		R	0.061	0.810	N/A[c]	
	f1	L	−0.112	0.657	−0.193	0.444
		R	0.042	0.868	N/A[c]	
	f2	L	0.290	0.243	−0.266	0.285
		R	0.053	0.836	N/A[c]	

Notes: [a)]Spearman rank correlation coefficient; [b)]Significant correlations uncorrected significance level $p < 0.05$ marked in **bold**; [c)]Lesion volume fraction for the right OR was 0 in all patients.

Abbreviations: AD – axial diffusivity; DWI – diffusion-weighted imaging; f2 – secondary partial volume fraction; FA – fractional anisotropy; L – left; LL – lesion load; M0 – visit at month 0 (baseline); M3 – visit at month 3; M6 – visit at month 6; OR – optic radiation; R – right.
